# TCMIO: A Comprehensive Database of Traditional Chinese Medicine on Immuno-Oncology

**DOI:** 10.3389/fphar.2020.00439

**Published:** 2020-04-15

**Authors:** Zhihong Liu, Chuipu Cai, Jiewen Du, Bingdong Liu, Lu Cui, Xiude Fan, Qihui Wu, Jiansong Fang, Liwei Xie

**Affiliations:** ^1^State Key Laboratory of Applied Microbiology Southern China, Guangdong Provincial Key Laboratory of Microbial Culture Collection and Application, Guangdong Open Laboratory of Applied Microbiology, Guangdong Institute of Microbiology, Guangdong Academy of Sciences, Guangzhou, China; ^2^Science and Technology Innovation Center, Guangzhou University of Chinese Medicine, Guangzhou, China; ^3^Division of Algorithm, Beijing Jingpai Technology Co., Ltd., Beijing, China; ^4^Research and Development Center, Guangdong Institute of Traditional Chinese Medicine, Guangzhou, China; ^5^Lerner Research Institute, Cleveland Clinic, Cleveland, OH, United States

**Keywords:** traditional Chinese medicine, database, cheminformatics, cancer immunotherapy, bioinformatics, immuno-oncology, medicinal herbs

## Abstract

Advances in immuno-oncology (IO) are making immunotherapy a powerful tool for cancer treatment. With the discovery of an increasing number of IO targets, many herbs or ingredients from traditional Chinese medicine (TCM) have shown immunomodulatory function and antitumor effects via targeting the immune system. However, knowledge of underlying mechanisms is limited due to the complexity of TCM, which has multiple ingredients acting on multiple targets. To address this issue, we present TCMIO, a comprehensive database of Traditional Chinese Medicine on Immuno-Oncology, which can be used to explore the molecular mechanisms of TCM in modulating the cancer immune microenvironment. Over 120,000 small molecules against 400 IO targets were extracted from public databases and the literature. These ligands were further mapped to the chemical ingredients of TCM to identify herbs that interact with the IO targets. Furthermore, we applied a network inference-based approach to identify the potential IO targets of natural products in TCM. All of these data, along with cheminformatics and bioinformatics tools, were integrated into the publicly accessible database. Chemical structure mining tools are provided to explore the chemical ingredients and ligands against IO targets. Herb–ingredient–target networks can be generated online, and pathway enrichment analysis for TCM or prescription is available. This database is functional for chemical ingredient structure mining and network analysis for TCM. We believe that this database provides a comprehensive resource for further research on the exploration of the mechanisms of TCM in cancer immunity and TCM-inspired identification of novel drug leads for cancer immunotherapy. TCMIO can be publicly accessed at http://tcmio.xielab.net.

## Introduction

The relationship between the human immune system and cancer can be traced back to 150 years ago, when Rudolf Virchow observed immune infiltration in human tumors and proposed the concept of mobilizing the immune system against cancer (Balkwill and Mantovani, 2001). Several decades later, American surgeon William Coley observed inflammatory responses and cancer clearance in some patients with bacterial infection (Coley, 1891). Due to the limited scientific understanding of the immune system, no great progress was made in the following century (Hoos, 2016). The milestone for cancer immunotherapy came with the identification of immune checkpoints, which control T-cell immune responses through on and off switches (Sharma and Allison, 2015). Immune checkpoint proteins maintain self-tolerance and prevent autoimmunity to protect tissues from damage by the immune system in a physiological situation. In cancer cells, the expression of checkpoint proteins can be dysregulated within the tumor microenvironment, an important factor in immune resistance (Hoos, 2016). Blockade of immune checkpoints seems to unleash the potential of the antitumor immune response in a fashion that is transforming human cancer therapeutics. Prominent checkpoint receptors are cytotoxic T-lymphocyte associated protein 4 (CTLA4), programmed cell death protein 1 (PD-1), and programmed death ligand 1 (PDL-1). In 2011, the first checkpoint inhibitor, ipilimumab, an anti-CTLA4 antibody developed by Bristol-Myers Squibb (BMS), was approved for treatment of melanoma (O’Day et al., 2010). This marked the beginning of the cancer immunotherapy revolution and changed the paradigm of anti-cancer drug development. Since then, various immunotherapies have been approved, and these new therapies have quickly become the standard of care for many cancer types (Pardoll, 2012).

Currently, most of the drugs developed in cancer immunotherapies are monoclonal antibodies that block T-cell checkpoint receptors and their cognate ligands. However, not all patients benefit from these drugs, and they can have adverse effects (Huck et al., 2018). To address these issues, a small-molecule approach has been proposed as an adjunct therapy, which is complementary to biologic drugs and shows a potentially synergistic effect (Huck et al., 2018). Small molecules can regulate the adaptive and innate immune system and ultimately influence the course of events within the tumor microenvironment. Small-molecule drugs show advantages over biological drugs, namely high feasibility, greater exposure within the tumor microenvironment, diverse formulation, and low cost (Adams et al., 2015). These advantages indicate enormous opportunities for small molecules in tumor immunotherapy (Adams et al., 2015).

Traditional Chinese medicine (TCM) has been widely used in China and has shown efficacy over a long history of clinical practice. Many herbs or ingredients from TCM have exerted immunomodulatory functions and antitumor effects via targeting the immune system (Ma et al., 2013). Ginsenosides, the effective ingredients of *Panax ginseng* C.A.Mey., have been one of the most extensively studied ingredients that enhance the host immune response effect. A direct effect of ginsenoside Rg1 on helper T-cell activity and on Th1/Th2 lineage development has been identified (Lee and Han, 2006). In addition, polysaccharides from *Ganoderma lucidum* (*Ganoderma lucidum* (Leyss.ex Fr.) Karst.)(Wang et al., 2017; Zhang et al., 2019), *Fructus psoraleae* (*Cullen corylifolium* (L.) Medik./Fabaceae) (Dai et al., 2007), *Brucea javanica* (*Brucea javanica* (L.) Merr./Simaroubaceae) (Dai et al., 2007), and *Radix Astragali* (*Astragalus mongholicus* Bunge/Fabaceae) (Jiang et al., 2010) were also reported to have immunological effects. Cancer patients can benefit from the immunomodulatory effects of TCM. A large retrospective cohort study found that patients with TCM utilization had a 32% decreased risk of death compared with patients without TCM utilization(Liao et al., 2017). These findings demonstrated that adjunctive therapy with TCM may improve overall survival for cancer patients.

The rapid development of immuno-oncology (IO) has led to increasing demand for informatics techniques for the analysis of IO targets, drugs, tumors, and the tumor microenvironment (Hammerbacher and Snyder, 2017). In order to track and understand the current IO agents in clinical development, the Cancer Research Institute presented an outline of the landscape of immuno-oncology drug development based on trusted and publicly available data sources (Tang et al., 2018b; Tang et al., 2018a; Xin Yu et al., 2019). The Cancer Immunome Atlas (TCIA) was developed, which aims to provide comprehensive immunogenomic analyses of next-generation sequencing data for solid cancers (Charoentong et al., 2017). Also developed was TIMER, a comprehensive resource for the systematical analysis of immune infiltrates across diverse cancer types (Li et al., 2017). These informatics resources provide comprehensive information on IO, which help the cancer research community with improving efficiency and with innovation.

**Figure 1:**
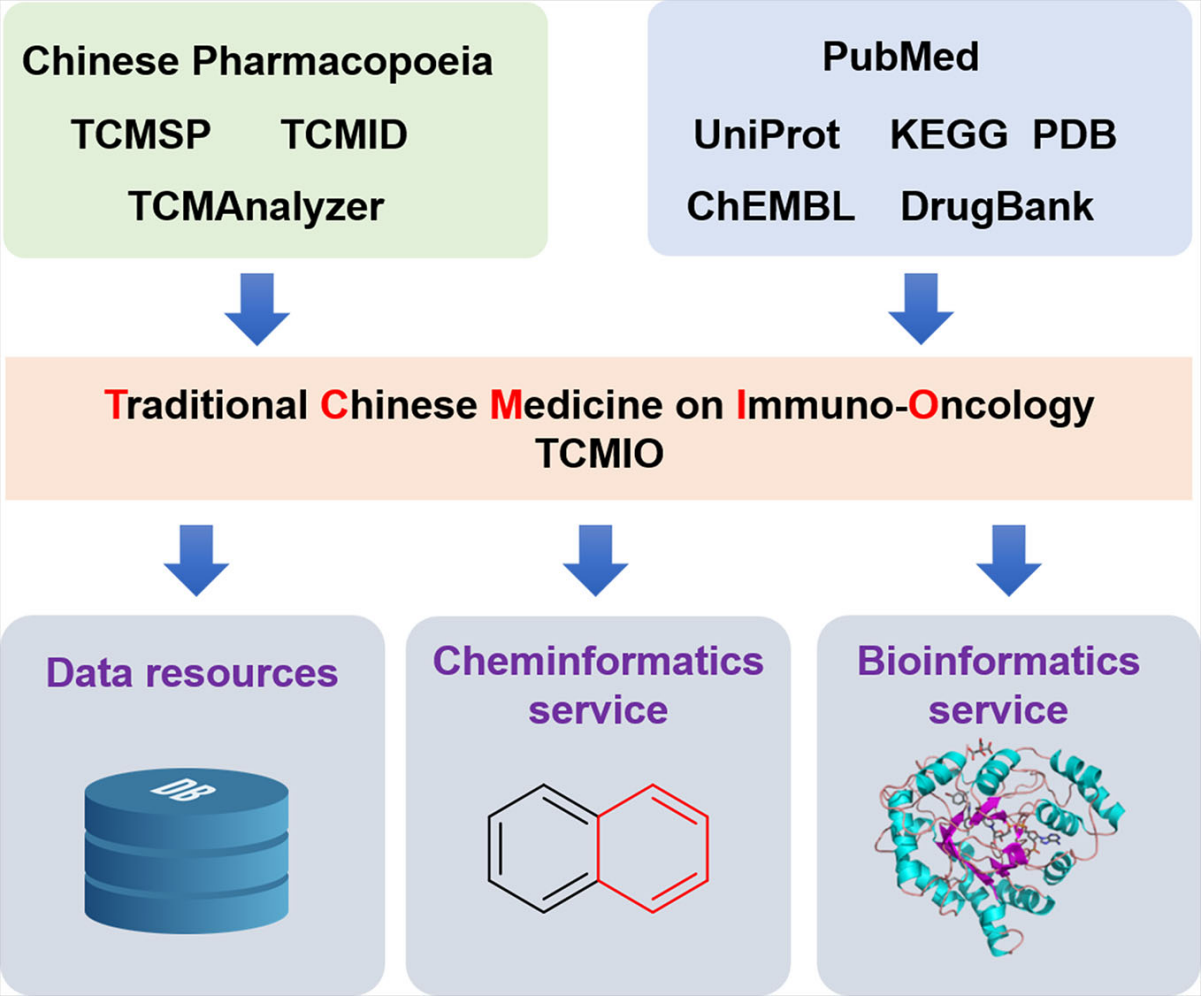
Overall architecture of the development of the TCMIO database.

Previous studies have illustrated the important roles of TCMs in immune regulation and have proposed a promising future for them in cancer immuno-therapies. However, to date, there has not been a comprehensive database of TCM for immuno-oncology. To address this challenge, we collected the IO targets and their small-molecule ligands, and information on those ligands was further mapped to the chemical ingredients of TCM. Comprehensive analysis of the relationship between TCM and cancer immunity was conducted. All these collected data were deposited in a web-based publicly accessible database, TCMIO, and cheminformatics and bioinformatics tools were integrated into the database for user analysis (**Figure 1**).

## Materials and Methods

### Data Preparation

The IO targets were extracted from the literature (Tang et al., 2018b; Tang et al., 2018a). For each target, the protein name and gene name were standardized using the public database UniProt (Bateman et al., 2015). The ligands for each target were extracted from ChEMBL (version 24.0) (Gaulton et al., 2017), with an activity threshold of 10 μM. The activity types only include Ki, Kd, IC_50_, EC_50_, and potency. The prescriptions and herbs were extracted from the Chinese Pharmacopoeia (version 2015). Kew Medicinal Plant Names Services (https://mpns.science.kew.org/mpns-portal/) were used to collect the species names of the TCM. The chemical ingredients were downloaded from TCMAnalyzer (Liu et al., 2018), which integrates data from TCMSP (Ru et al., 2014) and TCMID(Huang et al., 2018).

### Network-Based Inference Approach to Target Prediction

Since most TCM-derived natural products have few or no known IO targets, we exploited our in-house network-based predictive models to infer targets for each TCM ingredient. These models were developed to identify new targets of natural products via a balanced substructure-drug-target network-based inference (bSDTNBI) approach (Fang et al., 2017b), which could prioritize potential targets for known drugs and new chemical entities (NCEs) by resource-diffusion processes of the substructure drug-target network (Wu et al., 2016). During this process, two parameters, α and β, were imported to balance the initial resource allocation of different node types and the weighted values of different edge types. In addition, the parameter γ was also utilized to balance the influence of hub nodes. The final parameter values adopted for bSDTNBI were α = β =0.1, γ = –0.5, and k = 2. Among the four network models developed with different types of fingerprints, bSDTNBI_KR performed best, with the highest values of P (0.049), R (0.752), eP (27.02), eR (27.24), and AUC (0.959). Thus, bSDTNBI_KR was used to predict the top 50 targets for each TCM ingredient. Finally, we mapped all the predicted targets into manually curated IO targets to construct an herb–ingredient–target network.

### Cheminformatics Mining Tools

Chemical structure mining tools are required to investigate the chemical ingredients of TCM and the ligands of the IO targets. TCMIO integrates Bingo, which is one of the most popular modern cheminformatics application tools. Similarity, substructure, and full-structure searches are supported for mining the structures of TCM ingredients and ligands. Chemical similarity searching is based on the concept that structurally similar compounds usually having similar properties or biological activities (Maggiora et al., 2014). Similarity search estimates the similarity of the molecules by comparing their fingerprints using the default Tanimoto metric. Users can choose the appropriate similarity thresholds based on their requirements. A lower threshold will identify more targets but may bring lower accuracy, and vice versa.

### Bioinformatics Mining Tools

In order to investigate the mechanism of actions of TCM, gene enrichment analysis tools were added into TCMIO. The Database for Annotation, Visualization, and Integrated Discovery (DAVID) was integrated (Huang et al., 2009). DAVID provides a comprehensive set of functional annotation tools for understanding the biological meaning behind a list of genes. For TCM or a prescription, the experimentally validated or predicted IO target genes could be used as input. TCMIO uses the Python web service of DAVID to process the input genes and performs KEGG pathway enrichment analysis. For each pathway, the genes involved in the pathway, *P*-value, fold enrichment, Benjamini value, and false discovery rate (FDR) are provided.

### Web-Based Database Implementation

TCMIO is implemented as a web-based publicly accessible database that can be accessed through the major web browsers. Beego, an open-source framework for rapid web development in Golang, is used for backend services. RESTful APIs are provided in TCMIO, and detailed usage information and examples are described on a downloads page. All data are stored in a PostgreSQL open-source relational database (version 10.5). ipmDraw (http://ipmdraw.iprexmed.com) is used as a structure drawing tool. The ChemDoodle web component (Burger, 2015) is used for chemical structure visualization. Bingo (https://lifescience.opensource.epam.com/bingo) is used as the molecular search cartridge owing to the state-of-the-art indexing algorithms within its underlying database server and due to it supporting high-performance similarity, substructure, and full structure search functions. The tools used for constructing the TCMIO database are summarized in **Supplementary Table S1**.

## Results and Discussion

### Data Statistics

**Figure 2:**
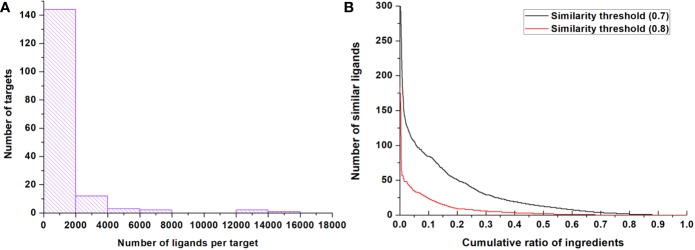
Data statistics. **(A)** The distribution of numbers of ligands for targets in ChEMBL. **(B)** The structurally similar ligands count against ingredients with different similarity thresholds (0.7 and 0.8); 20% of ingredients have at least 50 similar ligands with a threshold of 0.7 and 10 similar ligands with a threshold of 0.8.

After data preparation and standardization, a total of 400 unique IO targets derived from the literature were obtained, which can be classified into 34 protein families. TCMIO contains 157,195 ligand-target interactions, extracted from ChEMBL, connecting 126,973 ligands against 164 IO targets (164/400). There are an average of 958.5 ligands for each target (**Figure 2A**). In addition, a total of 1,493 TCM formulas (prescriptions) as well as 618 herbs collected in our previous work were also integrated into TCMIO. 16,437 unique TCM-derived ingredients were obtained, and their similarities against ligands were calculated. The result showed that 68% of herb ingredients are similar to at least one ligand using the similarity threshold value of 0.8. This percentage increases to 88% with the threshold value at 0.7. As shown in **Figure 2B**, 20% of ingredients have at least 50 similar ligands with a threshold of 0.7 compared to 10 with a threshold of 0.8. The structural similarity between chemical ingredients of TCM and the ligands of IO targets show promising prospects for TCMs and their ingredients in the development of immuno-therapy agents. The data entries in TCMIO are summarized in **Table 1**.

**Table 1:** Data statistics in TCMIO.

Type	Entries	Descriptions
Targets	400	Immuno-oncology targets derived from the literature and standardized using the UniProt database
Prescriptions	1493	Prescriptions extracted from the Chinese Pharmacopoeia (2015 Edition)
TCMs	618	TCMs extracted from the Chinese Pharmacopoeia (2015 Edition)
Prescription-TCM-Relations	13403	Prescription–TCM relations extracted from the Chinese Pharmacopoeia (2015 Edition)
Ligands	126972	Small molecule ligands against IO targets extracted from the ChEMBL database as an SDF file
Ingredients	16437	Ingredients of TCMs extracted from various public TCM databases
TCM-Ingredient-Relations	32847	TCM-ingredient relations extracted from public TCM databases
Ingredient-Target-Relations	41527	Ingredient–target (IO) relations based on network prediction
Ligand-Target-Relations	157195	Ligand–target (IO) relations extracted from the ChEMBL database

### Web Interface

#### Browse

**Figure 3:**
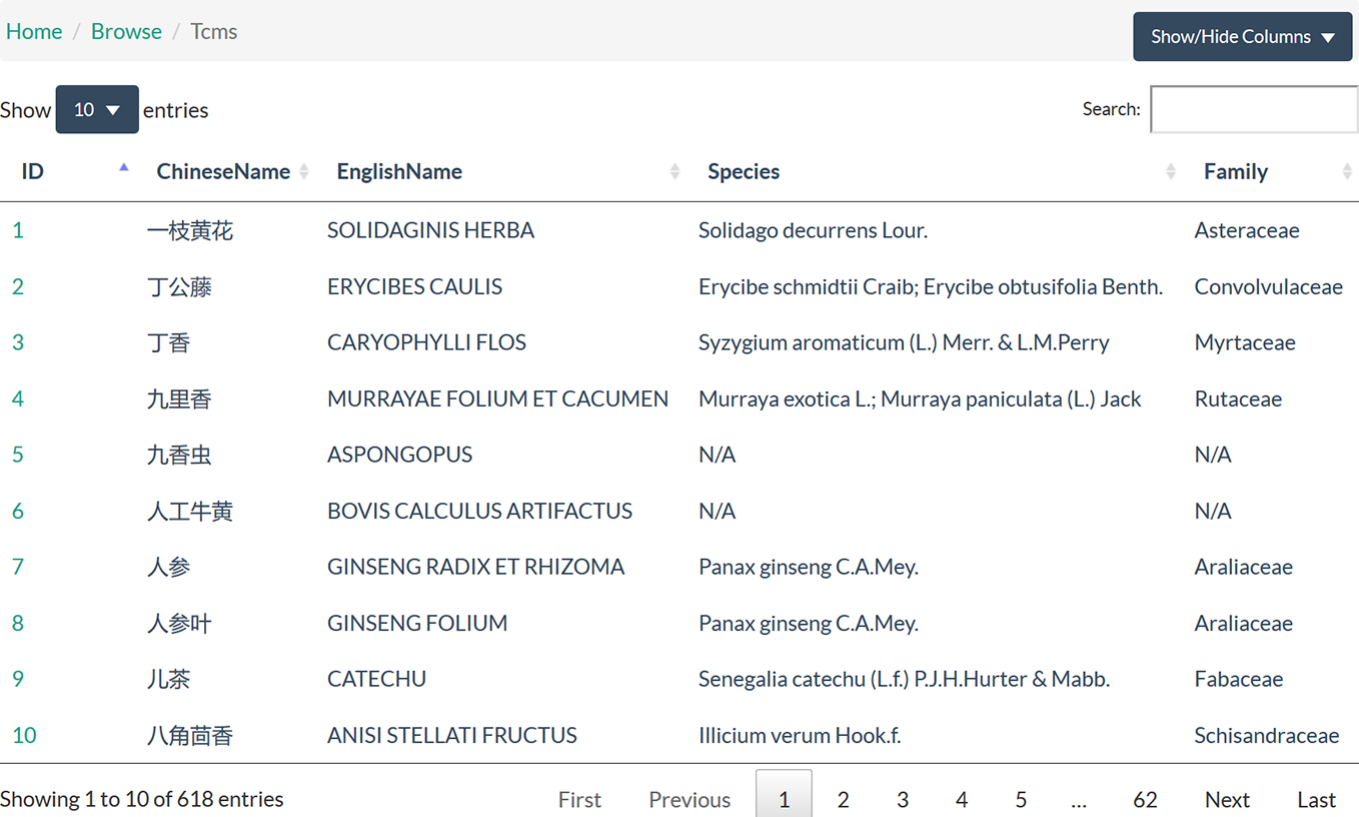
Snapshot of the browse page.

TCMIO provides interactive tables in which users can browse the targets, ligands, ingredients, herbs, and prescriptions. For each data entry, more information is provided in the details page. Meanwhile, public resource links are also provided for easy user access. For IO targets, the target IDs in ChEMBL (Gaulton et al., 2017), UniProt (Bateman et al., 2015), and KEGG(Kanehisa et al., 2017) are provided. Drug information for targets can be accessed through a DrugBank (Wishart et al., 2018) link. Structural information for targets can be accessed through a PDB(Rose et al., 2012) code. For ligands, the structure and the chemical properties, including molecular weight, hydrogen bond donor, hydrogen bond acceptor, and lipid-water partition coefficient AlogP, are given. For herbs, the part of the source used, property, flavor, channel tropism, effect, and indication are provided (**Figure 3**). For prescriptions, the TCM components, indication, and effect are given. The browse page provides comprehensive information in an easy to access format.

#### Structure

**Figure 4:**
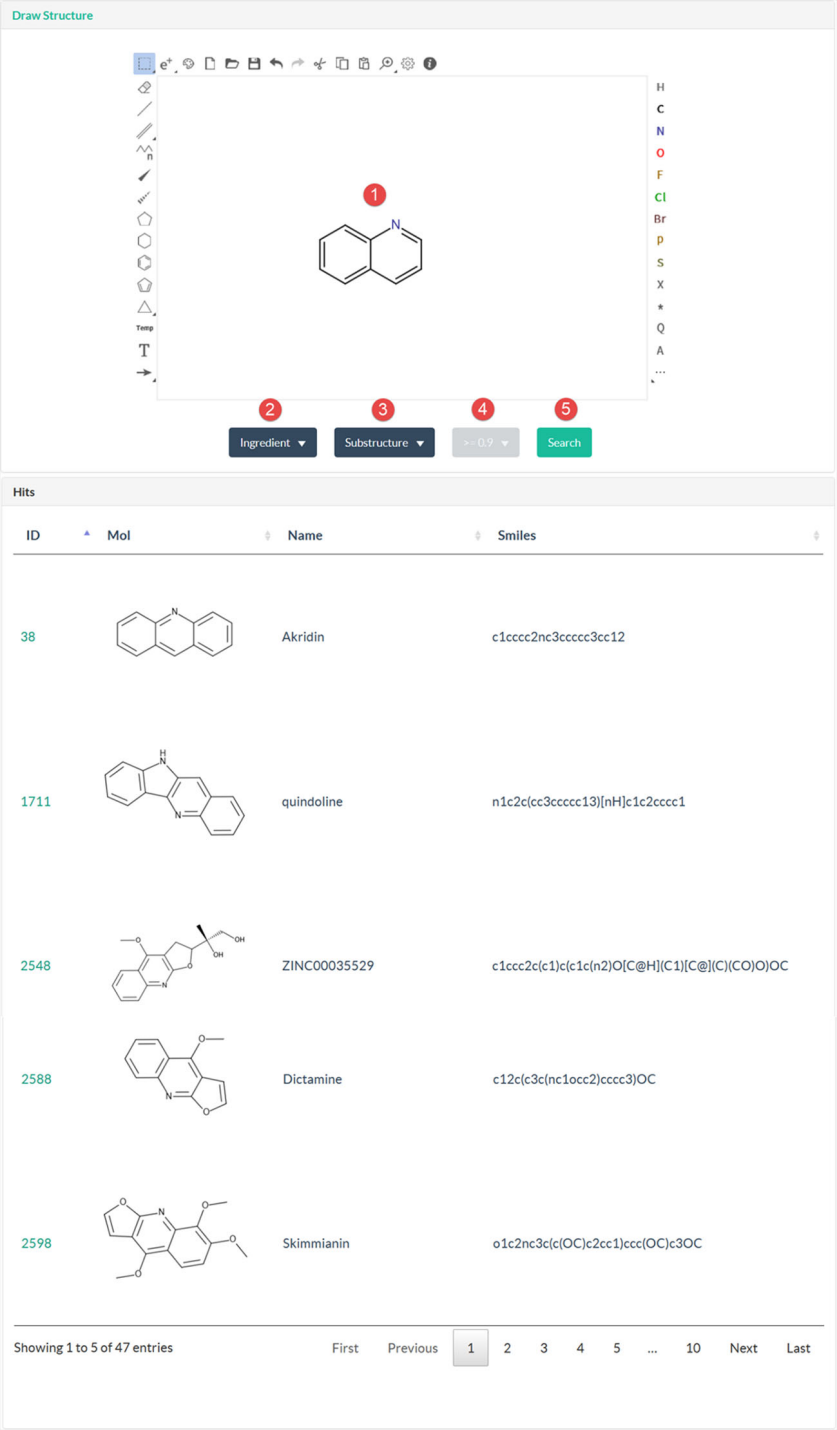
Snapshot of the structure page.

TCMIO aims to mine the relations of the TCM and IO through chemical structure mining tools. In the structure page, the user can conduct a structure search against ingredients or ligands. The widely used substructure and similarity search functions are provided. The user can draw a substructure or full structure of interest and then identify all the TCMs or targets with a structure query (**Figure 4**). All of the output TCMs share that query structure, which suggests that these TCMs may have the same biological functions. The output protein list is the potential IO targets of the query structure, suggesting the potential molecular mechanisms of this compound.

#### MOA

**Figure 5:**
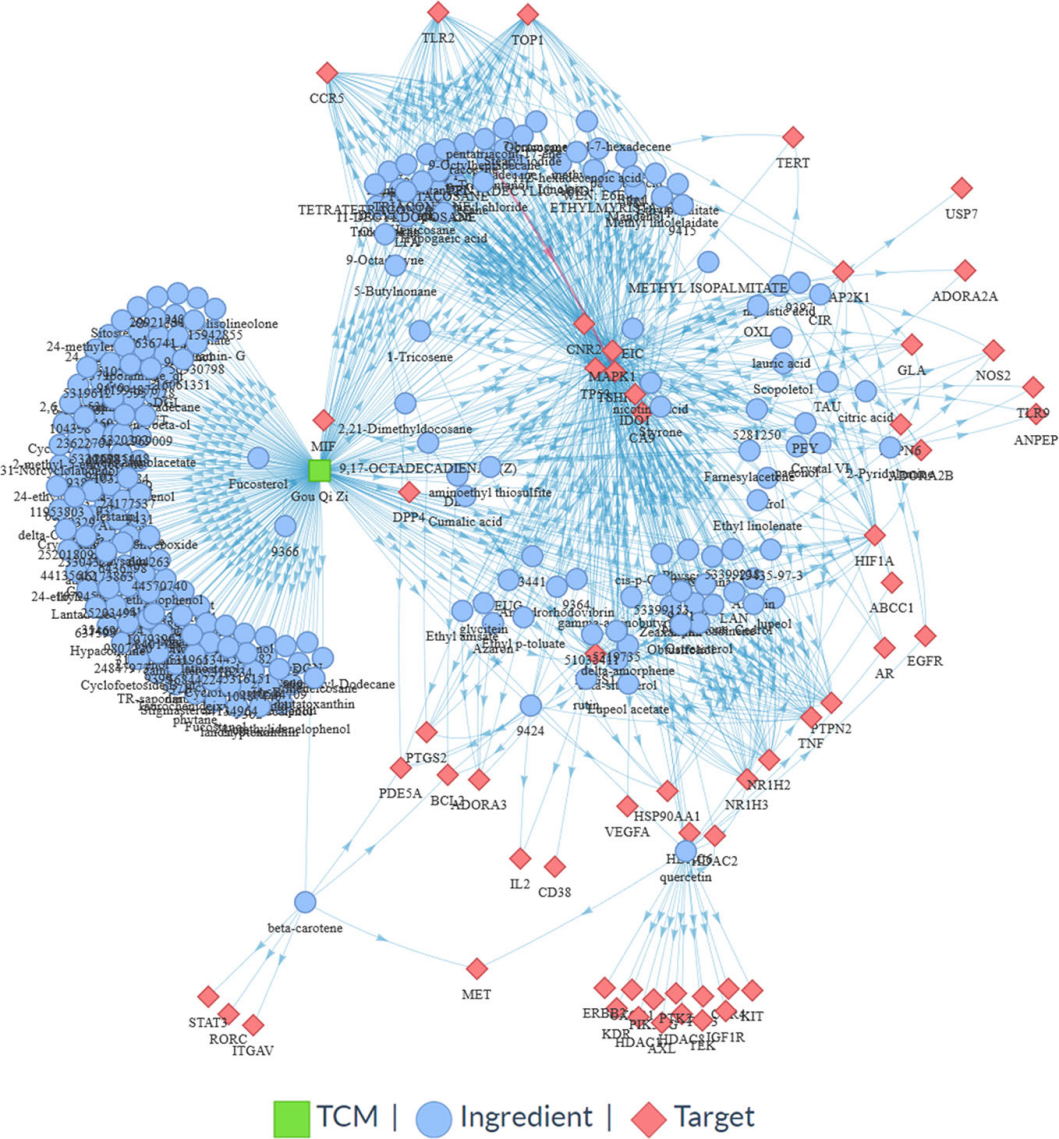
Snapshot of an herb (*Lycium barbarum* L.)–ingredient–target network on the MOA page.

**Figure 6:**
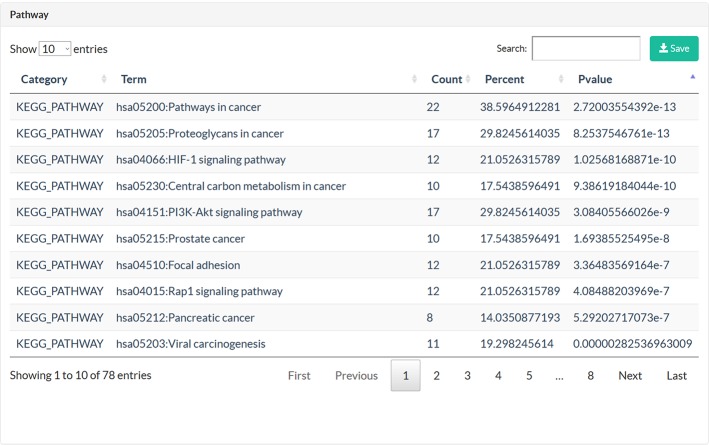
Snapshots of pathways on the MOA page.

TCMIO provides mechanism of action (MOA) exploration for TCMs in immuno-oncology based on the network pharmacology approach. On the MOA page, the user can submit herbs or prescriptions, and the ingredients and their target information,including the experimentally validated and predicted IO target using network-based approach. The targets are submitted to the DAVID web server for further gene enrichment analysis. Finally, TCMIO generates an herb–ingredient–target network (**Figure 5**) and interactive tables (**Figure 6**) for visualizing the relationships among them.

### Case Studies

In this section, we select a natural product (curcumin) and a Chinese herb (*Lycium barbarum* L.) as case studies to showcase how TCMIO facilitates research of TCM in immuno-oncology through target identification and MOA exploration.

#### Curcumin

Curcumin is a phenolic compound mainly isolated from the natural pleiotropic herb *Curcuma longa* L. Accumulating studies and clinical trials (https://clinicaltrials.gov) have demonstrated the potential therapeutic effects of curcumin against multiple tumors, such as breast cancer, prostate cancer, and pancreatic cancer (Devassy et al., 2015; Fang et al., 2017a). Recently, curcumin was identified as an effective immunomodulator to regulate the immunosuppressive tumor microenvironment (Liao et al., 2018; Wei et al., 2018). TCMIO has collected five known IO targets for curcumin from the ChEMBL database (**Table 2**). Besides, TCMIO allows users to identify potential IO targets of an input ligand through the substructure or fingerprint similarity search functions. Here, by setting the similarity >= 0.8, three new IO targets of curcumin are predicted, namely nuclear factor erythroid 2-related factor 2 (NFE2L2), prostaglandin G/H synthase 2 (PTGS2), and signal transducer and activator of transcription 3 (STAT3). Interestingly, a recent study has reported that curcumin can exhibit significant inhibitory activity on IL-6-induced STAT3 activation with an IC_50_ value of 1.6 µM, indicating its potential to be a candidate for the treatment of cancer related to the IL-6/STAT3 signaling pathway (Jang et al., 2019). The rest of the predicted targets provide novel potential proteins involved in cancer immunity by curcumin, which deserve to be further validated by experimental assays.

**Table 2:** Immuno-Oncology (IO) targets of curcumin identified by TCMIO.

Target Name	ChEMBL ID	Type	Activity (nM)
Epidermal growth factor receptor	CHEMBL203	Known	IC_50_ = 8650
Hypoxia-inducible factor 1-alpha	CHEMBL426	Known	Potency=6309.6
Mitogen-activated protein kinase 1	CHEMBL4040	Known	Potency=31622.8
Thyrotropin receptor	CHEMBL1963	Known	Potency=10000
Toll-like receptor 9	CHEMBL5804	Known	IC_50_ = 8362
Nuclear factor erythroid 2-related factor 2	CHEMBL1075094	Predicted	N/A
Prostaglandin G/H synthase 2	CHEMBL230	Predicted	N/A
Signal transducer and activator of transcription 3	CHEMBL4026	Predicted	N/A

#### Lycium barbarum L.

**Figure 7:**
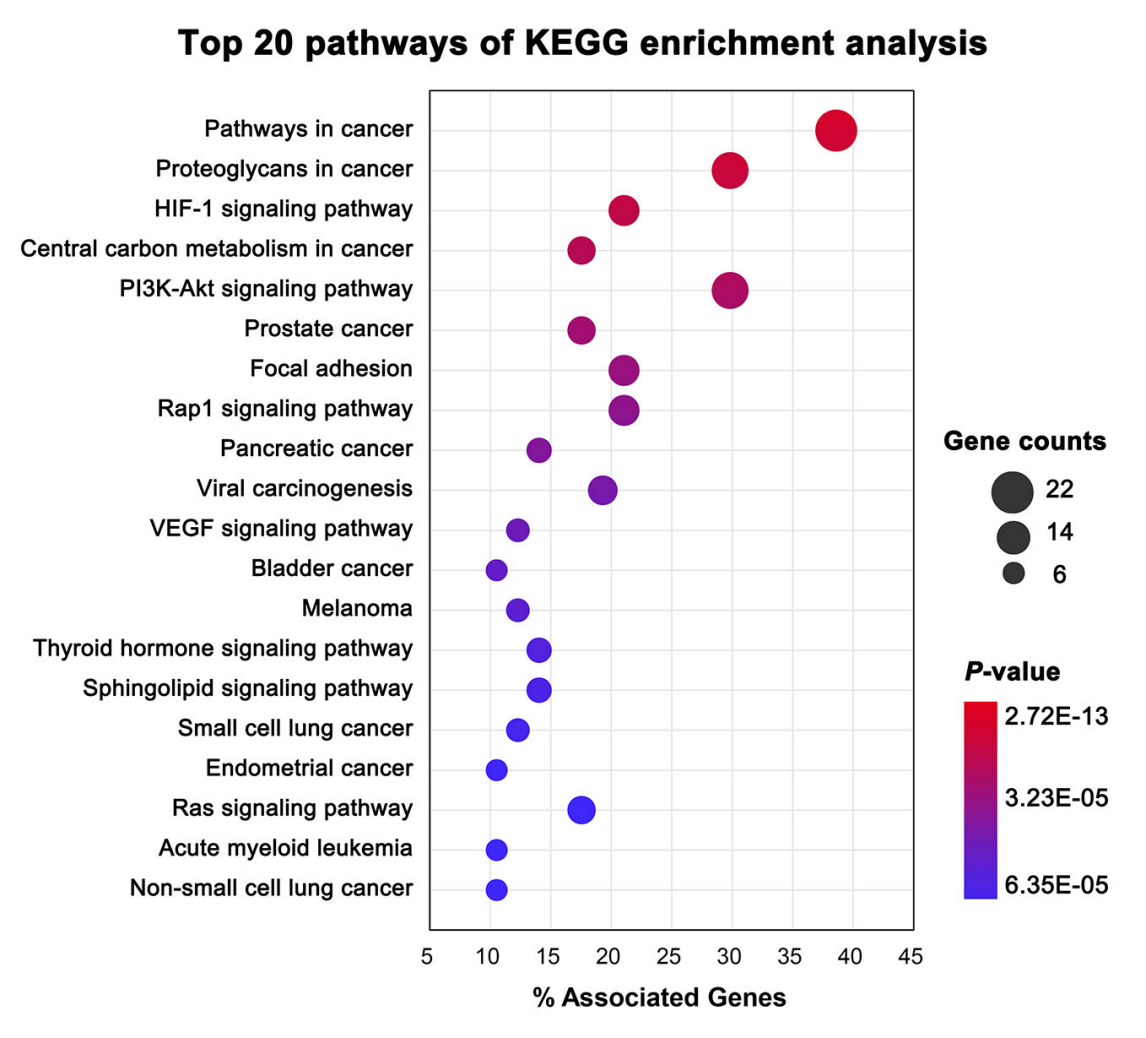
Top 20 pathways of KEGG enrichment with the lowest *P*-value for *Lycium barbarum* L., as obtained by TCMIO.

*Lycium barbarum* L., also known as *Wolfberry, Lycium chinense*, and *Goji berries*, is traditionally utilized in Asian as a medicinal herb for its poly-pharmacological qualities, including an antitumor effect (Tang et al., 2012). Numerous *in vivo* studies have confirmed that its main components, L. barbarum polysaccharides (LBPs), can enhance immunity and inhibit tumor growth (Cheng et al., 2015; Deng et al., 2018). However, the underlying mechanism of its antitumor immunity effect has not yet been fully elucidated. TCMIO integrates a useful systems pharmacology analysis function in the MOA page that provides convenient tools for uncovering the mechanisms of TCM in cancer immunity. As shown in **Figure 5**, the ingredient-target network of *Lycium barbarum* L. constructed by TCMIO consists of 761 interactions connecting 242 ingredients and 57 protein targets. KEGG enrichment analysis (**Supplementary Table S2**) indicates the high correlativity between *Lycium barbarum* L. and multiple cancers, such as prostate cancer (*P* = 1.69E-08), pancreatic cancer (*P* = 5.29E-07), and melanoma (*P* = 1.53E-05). Among the top 20 enriched pathways presented in **Figure 7**, the PI3K-Akt signaling pathway (*P* = 3.08E-09) is an important intracellular signaling pathway for regulating immune cell effector function and the tumor microenvironment(Collins et al., 2018; O’Donnell et al., 2018). Therapeutic inhibition of the PI3K-Akt signaling pathway could augment tumor immunosurveillance by preventing the activation of immunosuppression and enhancing antitumor immune-intrinsic properties (Xue et al., 2015). Besides, the VEGF signaling pathway (*P* = 6.28E-06) has been reported to exert modulatory effects on immune cells, including effector T cells, regulatory T cells (Tregs), myeloid-derived suppressor cells (MDSCs), tumor-associated macrophages (TAMs), and mast cells. Agents targeting the VEGF signaling pathway are becoming a promising therapeutic strategy for restoring antitumor immunity (Yang et al., 2018). Overall, the KEGG enrichment analysis performed by TCMIO suggests the potential cancer immunity pathways that may be mediated by *Lycium barbarum L*.

## Conclusion

Small molecules have shown great potential to stimulate the immune system to respond to various cancers. Widely used traditional herbs have displayed immunomodulatory functions and extensive antitumor effects, which indicates that exploring the relations of traditional herbs and immuno-oncology would expand our understanding of the MOAs of herbs and accelerate traditional medicine-inspired natural product drug discovery for cancer treatment. In this work, we developed a comprehensive database TCMIO, which integrates TCM data (prescription, herbs, and ingredients) and immuno-oncology data (IO targets and their small-molecule ligands). Moreover, cheminformatics and bioinformatics services were developed for data analysis. Various structure search methods are provided for mining the structures of traditional herbal ingredients and IO targeted ligands, which will help users to explore the herbs and design multi-target drugs. Herb–ingredient–target networks and pathway enrichment analysis for herb or prescription are provided for elucidating the MOAs of herbs. Currently, since the TCMs in TCMIO are only derived from the Chinese Pharmacopoeia (2015 Edition), many medicinal plants with immunomodulatory functions are not included here. This could be a future direction for TCMIO. To our knowledge, TCMIO is the first comprehensive database of herbs for use in immuno-oncology, and we believe that this database will bring benefits to the research communities of traditional herbs and immuno-oncology.

## Data Availability Statement

All datasets generated for this study are included in the article/**Supplementary Material**.

## Author Contributions

LX and JF contributed to the conception and design of the study. ZL and JD developed the web-based database. ZL, LC, BL, XF and QW collected and processed the data. ZL and CC performed data analysis. ZL, CC, and JF wrote the manuscript. All authors contributed to manuscript revision and read and approved the submitted version.

## Funding

This work has been supported by the National Natural Science Foundation of China (Grant No. 81703416, 81903912, and 81603318), the GDAS' Project of Science and Technology Development (Grant No. 2019GDASYL-0103009), the Youth Scientific Research Training Project of GZUCM (No. 2019QNPY05), and the Medical Scientific Research Foundation of Guangdong Province of China (Grant No. A2017071).

## Conflict of Interest

Author JD was employed by the company Beijing Jingpai Technology Co., Ltd.

The remaining authors declare that the research was conducted in the absence of any commercial or financial relationships that could be construed as a potential conflict of interest.
