# Supplementary material for: TCMIO: A Comprehensive Database of Traditional Chinese Medicine on Immuno-Oncology
Source: Front Pharmacol. 2020 Apr 15;11:439. doi: 10.3389/fphar.2020.00439 (PMC7174671; doi:10.3389/fphar.2020.00439)
Supplement: Supplementary file 1 [file DataSheet_1.docx]

**Supporting Materials**

**TCMIO: A Comprehensive Database of Traditional Chinese Medicine on Immuno-Oncology**

Zhihong Liu^1†^, Chuipu Cai^2†^, Jiewen Du^3†^, Bindong Liu^1^, Lu Cui^4^, Xiude Fan^5^, Qihui Wu^2^, Jiansong Fang^2,5^*, Liwei Xie^1,^*

^1^State Key Laboratory of Applied Microbiology Southern China, Guangdong Provincial Key Laboratory of Microbial Culture Collection and Application, Guangdong Open Laboratory of Applied Microbiology, Guangdong Institute of Microbiology, Guangdong Academy of Sciences, Guangzhou 510070, China.

^2^Science and Technology Innovation Center, Guangzhou University of Chinese Medicine, Guangzhou 510405, China

^3^Beijing Jingpai Technology Co., Ltd. 1500-1, Hailong Building Z-Park, Beijing 100090, China

^4^Guangdong Institute of Traditional Chinese Medicine, Guangzhou 510640, China

^5^Lerner Research Institute, Cleveland Clinic, Cleveland, OH 44195, United States

^†^These authors contribute equally.

**Table S1.** Tools used in constructing TCMIO database.

| **Tools** | **Purpose** | **Link** |
| --- | --- | --- |
| Chinese Pharmacopoeia (2015) | prescriptions and TCM data source | wp.chp.org.cn/front/chpint/en |
| UniProt | IO target information | [www.uniprot.org](http://www.uniprot.org/) |
| ChEMBL 24 | IO targets and ligands | www.ebi.ac.uk/chembl |
| KEGG | pathways data source | www.kegg.jp |
| TCMAnalyzer | ingredients of TCM | [www.rcdd.org.cn/tcmanalyzer](http://www.rcdd.org.cn/tcmanalyzer) |
| TCMSP | ingredients of TCM | lsp.nwsuaf.edu.cn/tcmsp.php |
| TCMID | ingredients of TCM | www.megabionet.org/tcmid |
| DAVID | GO enrichment analysis | david.ncifcrf.gov |
| ipmDraw | structure draw | ipmdraw.iprexmed.com |
| ChemDoodle Web | structure display of natural products | web.chemdoodle.com |
| Vis.js | network display | visjs.org |
| PostgreSQL | storage database | [www.postgresql.org](http://www.postgresql.org/) |
| Golang | web server language | golang.org |
| JQuery | foreground and background interaction | jquery.com |

**Table S2.** KEGG enrichment analysis result of *Lycium barbarum* L*.* (*P*-value < 0.05) by TCMIO.

| **Term** | **Count** | **%** | **P value** |
| --- | --- | --- | --- |
| hsa05200:Pathways in cancer | 22 | 38.59649 | 2.72E-13 |
| hsa05205:Proteoglycans in cancer | 17 | 29.82456 | 8.25E-13 |
| hsa04066:HIF-1 signaling pathway | 12 | 21.05263 | 1.03E-10 |
| hsa05230:Central carbon metabolism in cancer | 10 | 17.54386 | 9.39E-10 |
| hsa04151:PI3K-Akt signaling pathway | 17 | 29.82456 | 3.08E-09 |
| hsa05215:Prostate cancer | 10 | 17.54386 | 1.69E-08 |
| hsa04510:Focal adhesion | 12 | 21.05263 | 3.36E-07 |
| hsa04015:Rap1 signaling pathway | 12 | 21.05263 | 4.08E-07 |
| hsa05212:Pancreatic cancer | 8 | 14.03509 | 5.29E-07 |
| hsa05203:Viral carcinogenesis | 11 | 19.29825 | 2.83E-06 |
| hsa04370:VEGF signaling pathway | 7 | 12.2807 | 6.28E-06 |
| hsa05219:Bladder cancer | 6 | 10.52632 | 1.36E-05 |
| hsa05218:Melanoma | 7 | 12.2807 | 1.53E-05 |
| hsa04919:Thyroid hormone signaling pathway | 8 | 14.03509 | 2.48E-05 |
| hsa04071:Sphingolipid signaling pathway | 8 | 14.03509 | 3.27E-05 |
| hsa05222:Small cell lung cancer | 7 | 12.2807 | 4.30E-05 |
| hsa05213:Endometrial cancer | 6 | 10.52632 | 4.42E-05 |
| hsa04014:Ras signaling pathway | 10 | 17.54386 | 4.78E-05 |
| hsa05223:Non-small cell lung cancer | 6 | 10.52632 | 6.35E-05 |
| hsa05221:Acute myeloid leukemia | 6 | 10.52632 | 6.35E-05 |
| hsa05161:Hepatitis B | 8 | 14.03509 | 0.000109524 |
| hsa05214:Glioma | 6 | 10.52632 | 0.000130358 |
| hsa05142:Chagas disease (American trypanosomiasis) | 7 | 12.2807 | 0.000133342 |
| hsa05211:Renal cell carcinoma | 6 | 10.52632 | 0.000140225 |
| hsa05145:Toxoplasmosis | 7 | 12.2807 | 0.000181698 |
| hsa04520:Adherens junction | 6 | 10.52632 | 0.000198433 |
| hsa05140:Leishmaniasis | 6 | 10.52632 | 0.000198433 |
| hsa05220:Chronic myeloid leukemia | 6 | 10.52632 | 0.000212004 |
| hsa05206:MicroRNAs in cancer | 10 | 17.54386 | 0.000289238 |
| hsa05169:Epstein-Barr virus infection | 7 | 12.2807 | 0.000319562 |
| hsa05034:Alcoholism | 8 | 14.03509 | 0.000376257 |
| hsa04062:Chemokine signaling pathway | 8 | 14.03509 | 0.000507881 |
| hsa05160:Hepatitis C | 7 | 12.2807 | 0.000508154 |
| hsa04012:ErbB signaling pathway | 6 | 10.52632 | 0.000513126 |
| hsa04068:FoxO signaling pathway | 7 | 12.2807 | 0.000528859 |
| hsa04660:T cell receptor signaling pathway | 6 | 10.52632 | 0.000969494 |
| hsa05210:Colorectal cancer | 5 | 8.77193 | 0.001259197 |
| hsa04620:Toll-like receptor signaling pathway | 6 | 10.52632 | 0.001260329 |
| hsa05321:Inflammatory bowel disease (IBD) | 5 | 8.77193 | 0.001418019 |
| hsa05202:Transcriptional misregulation in cancer | 7 | 12.2807 | 0.00167006 |
| hsa05162:Measles | 6 | 10.52632 | 0.00341805 |
| hsa04914:Progesterone-mediated oocyte maturation | 5 | 8.77193 | 0.004360933 |
| hsa04640:Hematopoietic cell lineage | 5 | 8.77193 | 0.004360933 |
| hsa05144:Malaria | 4 | 7.017544 | 0.006197746 |
| hsa04915:Estrogen signaling pathway | 5 | 8.77193 | 0.006891743 |
| hsa05231:Choline metabolism in cancer | 5 | 8.77193 | 0.007391056 |
| hsa05146:Amoebiasis | 5 | 8.77193 | 0.008742673 |
| hsa04923:Regulation of lipolysis in adipocytes | 4 | 7.017544 | 0.00897913 |
| hsa04668:TNF signaling pathway | 5 | 8.77193 | 0.009031147 |
| hsa04931:Insulin resistance | 5 | 8.77193 | 0.009325795 |
| hsa05152:Tuberculosis | 6 | 10.52632 | 0.011278039 |
| hsa04020:Calcium signaling pathway | 6 | 10.52632 | 0.01180055 |
| hsa04210:Apoptosis | 4 | 7.017544 | 0.011861075 |
| hsa04722:Neurotrophin signaling pathway | 5 | 8.77193 | 0.01336109 |
| hsa04650:Natural killer cell mediated cytotoxicity | 5 | 8.77193 | 0.01412653 |
| hsa04664:Fc epsilon RI signaling pathway | 4 | 7.017544 | 0.015218261 |
| hsa04662:B cell receptor signaling pathway | 4 | 7.017544 | 0.015824754 |
| hsa04917:Prolactin signaling pathway | 4 | 7.017544 | 0.017078324 |
| hsa04320:Dorso-ventral axis formation | 3 | 5.263158 | 0.018076855 |
| hsa05216:Thyroid cancer | 3 | 5.263158 | 0.020705914 |
| hsa04810:Regulation of actin cytoskeleton | 6 | 10.52632 | 0.022123973 |
| hsa04550:Signaling pathways regulating pluripotency of stem cells | 5 | 8.77193 | 0.022285814 |
| hsa05143:African trypanosomiasis | 3 | 5.263158 | 0.026407145 |
| hsa04921:Oxytocin signaling pathway | 5 | 8.77193 | 0.02785024 |
| hsa05323:Rheumatoid arthritis | 4 | 7.017544 | 0.029936892 |
